# Suggestions on home quarantine and recovery of novel coronavirus patients

**DOI:** 10.2478/jtim-2023-0085

**Published:** 2023-07-05

**Authors:** Qiuyu Li, Chengyang Liu, Haiyun Li

**Affiliations:** Department of Respiratory and Critical Care Medicine, Peking University Third Hospital, Beijing 100191, China; Institute of Systems Biomedicine, School of Basic Medical Sciences, Peking University Health Science Center, Beijing 100191, China; Ministry of Education, Key Laboratory of Environment and Genes Related to Diseases, School of Basic Medical Sciences, Xi'an Jiaotong University, Xi'an 710049, Shaanxi Province, China; Department of Biochemistry and Molecular Biology, School of Basic Medical Sciences, Xi'an Jiaotong University, Xi'an 710049, Shaanxi Province, China

## Introduction

China has gradually relaxed its COVID-19 prevention and control regulations thanks to the efforts of the public at large.^[1,2]^ COVID-19 patients with no or mild symptoms are encouraged to stay at home for quarantine and recovery to reduce the transmission of the virus and avoid flooding the healthcare system. The World Health Organization advises those infected with or exposed to COVID-19 to isolate or quarantine at home in a separate bedroom and bathroom.^[3,4]^ Studies have shown that isolation could effectively reduce medical costs, economic damage due to work absenteeism and job loss, and the risks to and burdens on many families.^[5, 6, 7]^ However, patients may encounter questions about home isolation, healthcare, physical activities, and mental health. Here, we discuss and propose guidelines and recommendations on COVID-19 home quarantine and recovery, aiming to help these patients prevent disease spreading and self-manage their healthcare during the disease recovery.

## Suggestion on Home Quarantine

COVID-19 patients should stay at home and refuse all visitors. They should wear an N95 mask when entering the public area in the house. The door should be closed in each room, and the window should be open for ventilation for > 30 min at least twice daily. The central air conditioner should switch from circulation to fresh air. In the bathroom, windows should be opened, or the exhaust fan should be turned on. The room should be wet-cleaned and disinfected at least once a day. The toilet must be covered before flushing. The floor drains must be filled with water daily and covered by a lid. The lid can be held by a water-filled plastic bag or sealed with a silicone pad. If patients cough or sneeze, their mouth and nose should be covered by a paper tissue or plastic bag that must be sealed and discarded into a special trash can.^[8]^ The disease course in young and middle-aged patients is approximately seven days. Patients with prior COVID-19 vaccination tend to have a shorter disease course. The disease course in patients over 70 years old or with underlying diseases is more complicated.^[9,10]^ The home quarantine can be discontinued if the nucleic acid test Ct value is ≥ 35 on both the sixth and seventh days of isolation.

## Suggestion on Home Recovery

The academician Zhong Nanshan's team proposed the 4S principle of home exercises during the COVID-19 recovery, which are Simple, Safe, Satisfactory, and Save. The Simple refers to the easy method without assistance from professional staff. The Safe refers to the secured method without monitoring and assistance. The Satisfactory refers to the method acceptable to patients and medical staff.

The Save refers to cost-effectiveness. Meanwhile, various methods targeted at psychological support should be provided for patients and their families. Since COVID-19 infection primarily impacts the respiratory system, special attention should be given to patients in correct breathing methods to maximize respiratory function. A personalized recovery plan might be developed in patients with decreased physical fitness after evaluating the individual psychological status, cardiopulmonary function, and physical fitness.

### Patient education and symptomatic treatments

Patients should be educated on this disease, especially its effects on respiratory function. Adequate sleep ≥ 8 h can promote immune function, which can be achieved best during slow-wave sleep if they go to bed before 22:00.^[11]^ Patients with fever or pain can take antipyretics and analgesics, such as ibuprofen and paracetamol. In case of cough, compound fresh bamboo juice, Xuanfei Baidu Granules, and Jizhi Syrup can be used, or compound licorice and acetylcysteine if with excessive sputum. Huasu or Watermelon Frost lozenge can be tried when there is a severe sore or dry throat. Chlorpheniramine, Loratadine, Cetirizine, or Budesonide nasal spray can be used for stuffy and runny noses.

### Physical exercises, strength workouts, and disease-coping skills

Before the breathing exercises, fans should be placed at the door and windowsill, with the air blown into the room from the door and out of the room from the window. During the exercises, the patient faces the window with a fan blowing air from their behind. We review the following breathing exercises for reference. During these exercises, the patients can have standing, sitting, or semi-recumbent position when comfortable. The air is inhaled through the nose and exhaled through the mouth.

Exercises to improve dyspnea and reduce breathing workload. (1) Pursed lip breathing.^[12]^ With the shoulder and neck relaxed, the patient inhales slowly, then blows the air slowly through pursed lips three times a day, 3–5 min/time, and 5–10 breaths/time. (2) Position to relieve dyspnea. When there is dyspnea, the patient should find support in the standing position, then lean forward halfway, and adjust the breathing rate with pursed lip technique. The patient, if sitting, can lean forward halfway.^[13]^

Lung capacity improvement. (1) Thoracic expansion technique. Patients can place their hands in the chest area and apply slight resistance during inspiration. Patients should hold their breath for 0–3 sec (depending on the personal situation) at the end of inspiration, then slowly exhale the air. This can be performed three times/day, 3–5 min/time, and 5–10 breaths/time. (2) Necessary equipment. Patients take a deep inspiration and hold their breath for 0–3 sec (depending on the personal situation), then exhales the air slowly and fully through incentive spirometry,^[14]^ three times/day, 3–5 min/time, 5–10 breaths/ time. (3) Breathing exercises.^[13, 14, 15]^ a. Shoulder abduction exercise. The palms should turn inwards during the inspiration, and the shoulders should be abducted. During the expiration, the palms should turn outwards and slowly put down. b. Shoulder forwards bending exercise. The palms face downwards, and the shoulders are flexed forwards during the inspiration. Both hands are slowly put down during the expiration. These exercises can be performed twice daily and for ten breaths/time.

Breathing muscle strength improvement. The inspiratory muscle strength can be exercised through the inspiratory muscle training equipment.^[16]^ The patient can adjust the resistance during inspiration. This can be performed twice daily, 3–5 min/time, and 3–5 breaths/time.

The following is an example of aerobic exercise. The individual exercise plan should consult each own doctor. The exercises should begin with the easiest (heart rate is < 57% of maximum heart rate or increases by less than 30%, or exertion score is < 9/20) and be gradually adjusted based on the patient's cardiopulmonary exercise function. If there is no apparent discomfort during the easiest exercise and the blood pressure fluctuations do not exceed 20 mmHg, then the patient can start moderate exercises (heart rate is 57%–63% maximum heart rate or increases by 30%–39%, or the exertion score is 9–10/20) and vigorous exercises (heart rate is 64%–76% maximum heart rate or increases by 40%–59%, or the exertion score is 11–13/20) in one week.^[17]^ These exercises can be performed for 10–30 min/time. The first three and last five minutes are the warm-up and finishing stages. The patient can also perform stretching exercises (if moderate exercise is used, the accumulated exercise time is calculated). Continuous or intermittent steps, walking, treadmill, cycling, and traditional Chinese exercises such as Tai Chi, Baduanjin, and Wuqinxi can also be used.

Strength workout. Combining aerobic and resistance exercises can enhance cardiopulmonary function and muscle strength.^[18]^ The progressive resistance exercise method is recommended for strength training. Resistance can be achieved through self-gravity, elastic belts, dumbbells, and barbells. The four extremities and major muscles can be trained by the patient's own body weight. The core exercise can use hip, four-point support, or sit-ups. These exercises can be performed in each muscle group 2–3 times/week, 1–3 exercises/time, and 8–12 actions/exercise. The intensity range is gradually adjusted from the easiest (30%–49% incomplete rest method [IRM]) to moderate intensity (50%–69% IRM).^[17]^

Flexibility exercises can focus on the major muscle groups, such as the hamstring, quadriceps, and biceps brachii, as well as the muscle groups of the neck, shoulder, and trunk while emphasizing chest expansion. Each action is maintained for 15–20 sec with 1–2 groups/time. Dynamic traction, such as cat-cow stretch, arm stroke against the wall, and yoga, can also be used.

Exercise precautions. If a patient feels weak or in pain, the exercise intensity should be reduced, and its adjustment should be slowed down. The exercises should be held if shortness of breath, suffocation, or chest tightness occurs.

Occupational therapy program. (1) Energy conservation strategy. Patients may feel fatigued. The energy conservation strategy includes optimal organization, reasonable steps to coordinate the activities, simplified activities to reduce unnecessary movements, and effort-saving activities. (2) Relaxation. Abdominal breathing, meditation, and progressive muscle relaxation can reduce oxygen consumption and fatigue.^[19]^ (3) Home assistance. Patients and their families can work together on psychological issues. Online psychological support and self-management groups can improve their confidence. Professional advice should be obtained once there are psychological deteriorations. (4) Lifestyle redesign. Patients can participate in personalized lifestyle programs.^[20]^ Routine daily exercises, especially the upper extremity exercise, should be arranged to facilitate them to transit back to their previous activities.^[19]^ In these exercises, the patients can engage in favorable things to have a sense of achievement and happiness.^[21]^

### Mental health advises

Previous findings suggested that the COVID-19 pandemic causes negative consequences for mental health, especially in young adults. Home quarantine during COVID-19 has been associated with increased psychological distress and loneliness.^[22]^ Thus, appropriate risk communication and social contacts *via* social media or video calls are suggested to mitigate the negative effects of home quarantine on mental health.^[23]^

## Summary

We discuss and propose guidelines and recommendations on the home-based quarantine and recovery plan for COVID-19 patients. These guidelines and recommendations require further developments and modifications with better understanding and more evidence during our care for COVID-19 patients.
